# Valsalva maneuver: shortest optimal expiratory strain duration

**DOI:** 10.3402/jchimp.v1i2.7015

**Published:** 2011-07-18

**Authors:** Ramesh K. Khurana, Deepika Mittal, Norman H. Dubin

**Affiliations:** Department of Medicine, Union Memorial Hospital, Baltimore, MD, USA

**Keywords:** Valsalva maneuver, Valsalva ratio, expiratory strain, blood pressure, heart rate

## Abstract

**Purpose:**

To quantitate the level of difficulty and determine consistency of hemodynamic responses with various expiratory strain (ES) durations.

**Methods:**

Thirty-four healthy subjects performed the Valsalva maneuver (VM) with an ES duration of 10, 12, and 15 seconds in random order. Level of difficulty after each trial was rated 1 to 10, with 10 being the most difficult. Blood pressure and heart rate (HR) were recorded continuously and non-invasively. Parameters studied were Valsalva ratio (VR), early phase II (IIE), late phase II (IIL), tachycardia latency (TL), bradycardia latency (BL), and overshoot latency (OV-L). Consistency of responses was calculated.

**Results:**

Difficulty increased significantly with increased ES duration: 5.1±0.1 (mean±SEM) at 10 seconds, 5.9±0.1 at 12 seconds, and 6.8±0.1 at 15 seconds (*p*<0.001). Phase IIE, TL, BL, OV-L, and VR response did not differ statistically with increasing ES durations, and there were no differences in variability. Phase IIL response increased significantly with increasing ES duration. Phase IIL was poorly delineated in 14 of 102 trials with 10 seconds ES duration.

**Conclusions:**

ES duration of 10 seconds created a low level of difficulty in healthy individuals. This strain duration produced consistent hemodynamic response for all parameters tested except IIL phase. The absence of IIL phase with 10 seconds ES should not be interpreted as an indicator of sympathetic vasoconstrictor failure.

The Valsalva maneuver (VM) is an established non-invasive quantitative test for the measurement of cardiovagal, cardiovascular sympathetic, and baroreflex functions (1). It is a commonly employed clinical tool to assess the integrity of autonomic cardiovascular functions in diseases of major public health significance such as diabetes mellitus.

Since 1966, the quantification of heart rate (HR) changes during VM has been used as a simple, reproducible method to assess cardiac and autonomic functions (2, 3). The Valsalva ratio (VR), the ratio of maximal to minimal HR during the VM, was previously the most commonly employed index. The advent of instruments capable of recording blood pressure (BP) non-invasively and continuously (Finapres, Colin instrument) has made it possible to monitor both BP and HR simultaneously, allowing development of new criteria based on BP changes for evaluating sympathetic function, such as quantification of VM late phase 2 and phase 4 (4). Continuous BP and HR recordings and computerization of data have also made it possible to measure latency values between points on the expiratory strain (ES) graph and on BP and HR curves, thus allowing the creation of time change-dependent indices such as tachycardia latency(TL), bradycardia latency(BL), and overshoot latency (5).

For consistency and reproducibility, hemodynamic responses to the VM depend upon adherence to various technical variables including ES magnitude and duration (4). The ES duration, an important confounding variable, has varied from 10 seconds to 45 seconds in various studies (2, 4–7). Benarroch and colleagues described a 15-second ES duration as a practical optimum (8). However, there is wide variation in the ability of patients to maintain ES beyond a few seconds. The clinician is compelled to analyze abridged VM for diagnostic purposes. To improve the practical utility of VM, we decided to study VMs of varying ES durations for their ease and clinical usefulness.

The goals of this prospective study were to quantitate the level of difficulty and to study intrasubject variation in healthy subjects to determine the least difficult and shortest ES duration that provides the most consistent hemodynamic responses. The effect of ES duration on the VR and newer parameters of BP phases, BL, TL, and overshoot latency(OV-L) were evaluated (2, 4, 5).

## Methods

### Subjects

The study was approved by the Institutional Review Board. All subjects gave informed consent. Thirty-four subjects were recruited through hospital bulletin board notices, from house officers, and from the local community. Mean age was 24 years (range, 22 to 31 years) with 18 males and 16 females. All were normotensive. None had a history of cardiovascular, respiratory, or neurologic disease, and none were taking any medication at the time of this study. Subjects who had a history of headaches induced or aggravated by cough were excluded.

### Equipment and data acquisition

The mouthpiece (W.R. Electronics Co, Stillwater, MN) consisted of a small chamber with three orifices, one end for the attachment of a disposable mouthpiece, the other end for the replaceable insert for oral leak, and a side outlet for a tube that connected to a transducer for recording ES and to a mercury manometer for displaying expiratory pressure.

Blood pressure was recorded continuously and non-invasively by Ohmeda Finapres 2300 (Louisville, CO), a photoplethysmographic device. An appropriate-size finger cuff was used and the left hand was supported in a sling to keep the hand at heart level. Heart rate was recorded continuously from a precordial electrocardiograph (lead II) using a Hewlett-Packard HR monitor. Expiratory pressure during VM was monitored via a parallel transducer, and the data were collected using Biopac Student Lab Pro.

### Study design

The participants were asked to refrain from smoking and from consuming coffee or alcohol on the day of testing. All recordings were made in a quiet lab with an ambient temperature of 22–23°C. The procedure was performed with subjects in a supine position and the head propped on a small pillow. The subject was connected to the instruments and given two or three practice runs. The VM was initiated at the end of normal inspiration. Care was taken that the expiratory pressure rose sharply at the onset, was maintained at 40 mmHg for the duration of the strain, and fell abruptly at the termination of strain. The manometer, positioned in front of the subject, provided visual feedback of expiratory pressure. The duration of strain was carefully timed by a stopwatch. The subject was asked to breathe normally at the end of a maneuver avoiding deep inhalation or exhalation. At least 3 min of recovery time was allowed after each VM(2, 4). Three different VM durations commonly used in clinical autonomic laboratories were tested in random order: 10, 12, and 15 seconds. The subjects rated the level of difficulty after each trial on a scale of 1 to 10, with 10 being the most difficult.

### Measurements and analysis

Systolic blood pressure (SBP), diastolic blood pressure (DBP), mean blood pressure (MBP), HR, and ES (duration and amplitude) were displayed simultaneously during the VM. Phase-to-phase changes in beat-to-beat BP and HR, time intervals between the ES efforts, and points on the BP and HR curve were used for calculating various parameters.

The normal cardiovascular response to VM is comprised of four sequential phases. At the onset of forced expiration in phase 1, there is an abrupt and transient rise in SBP and DBP with a variable presence of one to two beats of bradycardia. Phase 2, during continued strain, is subdivided into early and late components. The early component shows a fall in SBP and DBP accompanied by tachycardia. In late phase 2, BP shows a compensatory increase. Phase 3 is characterized by a brief and abrupt decline in BP accompanied by further tachycardia following termination of ES. Phase 4 displays a sustained increase in SBP (systolic overshoot) and DBP accompanied by bradycardia (4). We used VR, a traditional parameter, and five newer, probably more sensitive, parameters based on the BP curve, its relationship to ES stimulus, and HR responses.

1. VR: ratio of maximum HR during VM divided by lowest HR within 30 seconds of termination of VM (2)
2. Early phase II (IIE, mmHg): difference in blood pressure from baseline to trough of phase II (4)
3. Late phase II (IIL, mmHg): difference in DBP between end of phase IIE to the beginning of phase III (4)
4. Tachycardia latency (TL): measured from point of lowest BP in VM phase 3 to highest HR of induced tachycardia (5)
5. Bradycardia latency (BL): measured from point of highest BP in VM phase 4 to lowest HR of subsequent bradycardia within 30 seconds of VM (5)
6. Overshoot latency (OV-L): measured from end of VM to the peak of systolic pressure overshoot, defined as BP reading exceeding the baseline, within 30 seconds after VM (5)

Visualization of all phases of cardiovascular response to VM was required to calculate parameters in our analysis. The data were collected from 30 seconds before the test until 30 seconds after the test. Delineation of the four expected phases of BP responses to the VM was evaluated, and poorly delineated responses were omitted from analysis. Baseline supine SBP, DBP, MBP, and HR averages were determined during a 30-second interval directly preceding the VM. All six parameters were calculated for each VM. For each VM duration, three trials were performed and the results of the three trials were averaged. Data were compared using analysis of variance (ANOVA). Post hoc analysis by Tukey's test was done if differences were statistically significant (*P* < 0.05). All data were expressed as mean±SEM. To test for variability, the standard deviation (SD) for each individual's three trials was determined and individual coefficient of variation (CV) was calculated (CV = SD/mean) and expressed as a percentage. The mean CVs for each ES duration were determined and compared by ANOVA. The level of difficulty data for each trial was tabulated, and difficulty scores were compared between the different expiration times by ANOVA with repeated measures.

## Results

Mean difficulty score was 5.1±0.1 (mean±SEM) at 10 seconds, 5.9±0.1 at 12 seconds, and 6.8±0.1 at 15 seconds ES duration (*P*<0.001). Post hoc analysis showed all means were significantly different from each other. Difficulty level of seven or higher, considered to represent severe difficulty, was reported by 7 subjects (21%) at 10 seconds, 15 subjects (44%) at 12 seconds, and 19 subjects (56%) at 15 seconds ES duration. The highest reported difficulty level was 8 at 10 seconds, 9 at 12 seconds, and 10 at 15 seconds ES duration.

**Fig. 1:**
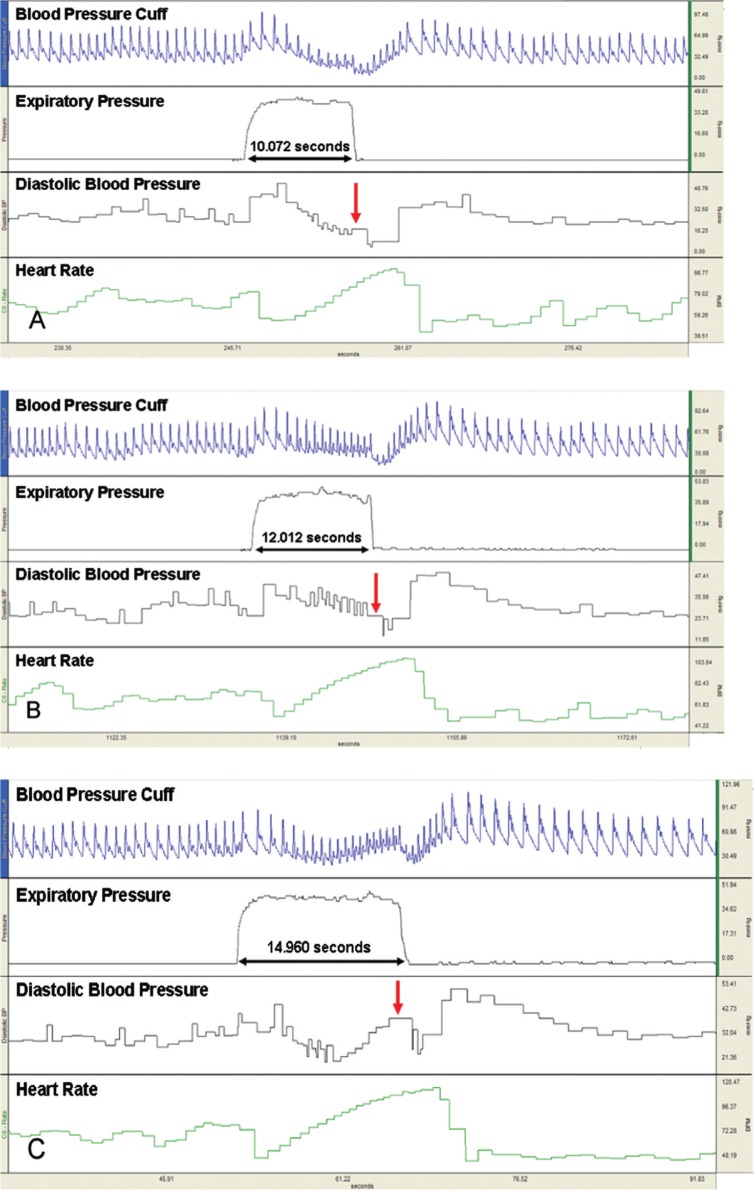
Valsalva maneuver pressure pattern showing poor to absent delineation of late phase II (vertical arrow) with 10-second (A) and 12-second (B) maneuver and presence with 15-second expiratory strain (C).

Phase IIL (DBP) of VM was poorly delineated (Fig. 1) in 14 of 102 trials with 10 seconds, 8 of 102 trials with 12 seconds, and zero of 102 trials with 15 seconds ES duration. These trials were not included in the analysis. The VR, DBP IIE, TL, BL, and OV-L did not differ statistically with increasing ES durations. Further, the amount of variability was consistent as determined by comparing CVs among strain durations. The DBP response of IIL phase displayed a statistically significant increase with increasing ES duration (Table 1). Post hoc analysis showed all means were significantly different from each other.

**Table 1:** Statistical analysis of cardiovascular responses to varying expiratory strain durations (mean±SEM)^a^

Parameter	Expiratory strain duration (s)				
		
	10	12	15	*P*–value for means	*P*–value for CVs^b^
Difficulty	5.1±0.1	5.9±0.1	6.8±0.1	<0.001*	NA
Valsalva Ratio	1.86±0.04	1.95±0.04	2.0±0.04	0.07	0.62
DiastolicBloodPressure IIE	0.80±1.33	−0.97±1.19	−1.75±0.92	0.28	0.98
DiastolicBloodPressure IIL	8.58±0.44	11.80±0.62	17.27±0.85	<0.001*	0.23
Bradycardia latency	5.63±0.47	5.64±0.45	5.39±0.44	0.91	0.33
Tachycardia latency	2.32±0.14	2.23±0.16	2.10±0.09	0.54	0.71
Overshoot latency	8.19±0.36	7.37±0.30	8.02±0.44	0.26	0.51

a CV, coefficient of variation; NA, not applicable because difficulty was assessed only once per duration

* Statistically significant (*p*<0.05).

b *P*–value for CV was calculated by comparing the CVs using analysis of variance for the three ES durations tested. No CV *P*-values were significant, indicating there were no significant differences in variation in measurements for the three durations tested.

## Discussion

These data show that, in young healthy subjects, a VM ES duration of 10 seconds is associated with the lowest difficulty level and provides consistent hemodynamic response except for IIL DBP phase. Patients with autonomic dysfunction are expected to have even more difficulty in sustaining high ES for a long period (8). The findings suggest that, with the exception of DBP phase IIL, all hemodynamic criteria measured in this study will be consistently visualized in patients at the lowest tested ES duration.

The VM, a neurocardiovascular reflex with afferent, central, and efferent pathways, involves both cardiovagal and sympathetic outflows. There are differences in the latency of cardiovagal and sympathetic responses due to differences in neuroeffector delays involving interneurons in central processing and due to differences in the length and conduction velocities of the efferent nerves. The efferent vagal limb is shorter and conducts more rapidly than the sympathetic limb. Conduction velocity in efferent cardiac vagal fibers is about 7 m/second, whereas in muscle sympathetic fibers it is about 1 m/second (9). A minimum of 7 to 8 seconds ES duration is necessary for the onset of HR and blood pressure responses (3, 8, 10, 11). The maximum optimal ES duration is debated. Korner and colleagues state that in most studies, ES duration has been kept short (15 to 20 seconds) in relation to the time constant of the sympathetically mediated responses. A 30-second ES duration is probably necessary to achieve circulatory stability (6). The functional magnetic resonance imaging (fMRI) of the brain in 12 subjects who ‘exhaled strongly into the mouthpiece for 18 seconds’ demonstrated the following signal intensity peaks: hippocampus and amygadala (5.4±1.6 seconds), brainstem (∼14 seconds), cerebellum (∼15 seconds), insular cortex (14±1 seconds), and the lentiform nuclei and the lateral prefrontal cortex (∼21 seconds) (12). The fMRI study indicates that central processing time for VM-induced hemodynamic responses may be longer than previously believed. These scientific observations indicate the need for a minimum of 7 to 8 seconds and a maximum of 30 seconds of ES to provide adequate stimulus for circulatory response. The duration of ES required for patients with diseases affecting afferent or efferent pathways may be even longer (10, 11, 13). The current data suggest that normal subjects have difficulty sustaining ES of 15 seconds. Patients with autonomic disorders are likely to encounter even more difficulty than normal subjects.

Benarroch and colleagues studied ES duration of 5, 10, 15, or 20 seconds in 20 subjects at 20 and then at 50 mmHg ES magnitude (8). The four phases of VM were clearly identified in all subjects at 5, 10, 15, and 20 seconds ES duration. These investigators observed a low correlation between the HR and BP changes and the duration of VM except for maximal increase in MAP in IIL and IV phases (8). In the current study, 10 seconds of ES duration was sufficient for eliciting consistent VR due presumably to faster conduction velocity of vagal fibers. Bradycardia and tachycardia latencies were not significantly affected by increased ES duration. However, the influence of ES duration on sympathetic function appeared to be parameter specific. Overshoot latency was not significantly affected by shorter duration, whereas IIL phase of BP was difficult to delineate with 10 seconds ES duration in approximately 14% of trials. The current findings support those of Benarroch et al (8) in further quantifying that 10 seconds of ES duration is clinically useful to provide consistent hemodynamic responses in all parameters measured except DBP IIL.

Because the absence of IIL phase with 10 seconds ES is not unusual, it should not be interpreted as an indicator of sympathetic vasoconstrictor failure.
